# Synthesis of flower-like magnetite nanoassembly: Application in the efficient reduction of nitroarenes

**DOI:** 10.1038/s41598-017-09477-7

**Published:** 2017-09-14

**Authors:** Kasibhatta J. Datta, Anuj K. Rathi, Pawan Kumar, Josef Kaslik, Ivo Medrik, Vaclav Ranc, Rajender S. Varma, Radek Zboril, Manoj B. Gawande

**Affiliations:** Regional Centre of Advanced Technologies and Materials, Department of Physical Chemistry, Faculty of Science, Palacký University, Šlechtitelů 27, 783 71 Olomouc, Czech Republic

## Abstract

A facile approach for the synthesis of magnetite microspheres with flower-like morphology is reported that proceeds *via* the reduction of iron(III) oxide under a hydrogen atmosphere. The ensuing magnetic catalyst is well characterized by XRD, FE-SEM, TEM, N_2_ adsorption-desorption isotherm, and Mössbauer spectroscopy and explored for a simple yet efficient transfer hydrogenation reduction of a variety of nitroarenes to respective anilines in good to excellent yields (up to 98%) employing hydrazine hydrate. The catalyst could be easily separated at the end of a reaction using an external magnet and can be recycled up to 10 times without any loss in catalytic activity.

## Introduction

The selective reduction of nitroarenes has attracted a great deal of attention as the resulting anilines are important intermediates for the manufacture of pharmaceuticals, dyes, polymers, and fine chemicals^1–3^. Generally, the synthesis of anilines entails catalytic^4–6^ and non-catalytic methods employing different reducing agents^7–9^. The non-catalytic processes use either Bechamp or sulphide reduction technology which generates large amounts of undesirable waste that is detrimental to the environment^10^. On the other hand, catalytic process is a well-established technology but relies on mainly expensive precious metal catalysts, namely Pd, Pt, and Ru which lack chemoselectivity in the presence of other common reducible functional groups^6, 11–14^. Furthermore, when hydrogen is used as the reducing agent, high temperature and pressure are usually needed with requirement of the specialized equipment. These limitations can be circumvented using various hydrogen donors such as formic acid^4, 5^, hydrazine hydrate^9, 15–19^, ammonium salts^20^, and sodium borohydride^21^, among others, in presence of various metal catalysts.

Amongst hydrogen donors, hydrazine monohydrate is particularly noteworthy as it produces only harmless by-products, such as nitrogen gas and water, and is relatively safe and easy to handle compared to its anhydrous form. From a sustainability perspective, substitution of precious metals by earth-abundant base metals is a highly desirable pursuit for heterogeneous catalysis. In this aspect, magnetic materials especially iron-based catalysts in organic synthesis have received significant attention, as iron is plentiful, cost effective, and relatively ﻿﻿﻿environmentally benign element^22–27^. Consequently, it is not surprising that the reduction of nitroarenes has been reported utilizing a combination of hydrazine and iron catalysts namely various iron salts, its complexes, and oxide forms and as a supported catalysts^9, 15, 17–19, 28, 29^. The readily available magnetic iron oxide nanoparticles stand out to be very attractive candidates as they are cost-effective nanocatalysts;^30–36^ being recoverable effortlessly with an external magnet due to the paramagnetic behaviour thus avoiding cumbersome filtration/separation processes^12, 29, 37–39^. This strategy could significantly improve the catalytic efficiency and decrease the operational cost which is crucial for practical applications.

**Figure 1:**
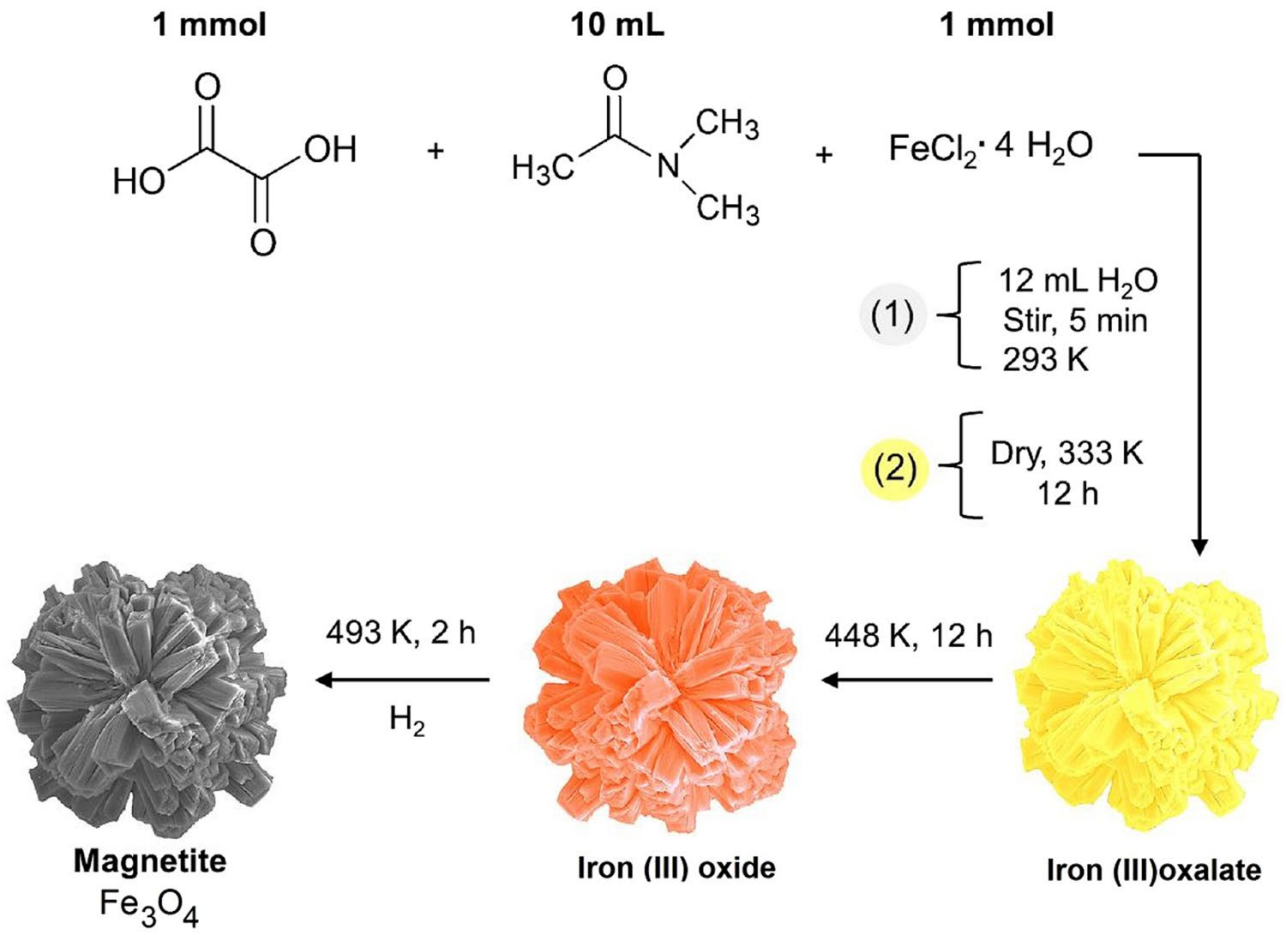
Schematic illustration of the synthesis of Fe_3_O_4_ nanoflower.

Herein, we report a simple approach for the synthesis of magnetite *via* thermally induced solid state reaction of iron (III) oxide under hydrogen a atmosphere (Fig. 1). It is interesting to note that the flower/rod like morphology of the precursor is well preserved even after the hydrogen treatment. The as-prepared magnetite catalyst is characterized by several techniques, namely XRD, FE-SEM, TEM, nitrogen adsorption-desorption isotherm and Mössbauer spectroscopy. The magnetite acts as a catalyst for the transfer hydrogenation of nitroarenes with hydrazine hydrate as the reducing agent in a microwave reactor affording nearly quantitative yields. The salient features of this work are the excellent catalytic performance, simple procedure, easy separation, and the excellent reusability of the catalyst.

## Results and Discussion

**Figure 2:**
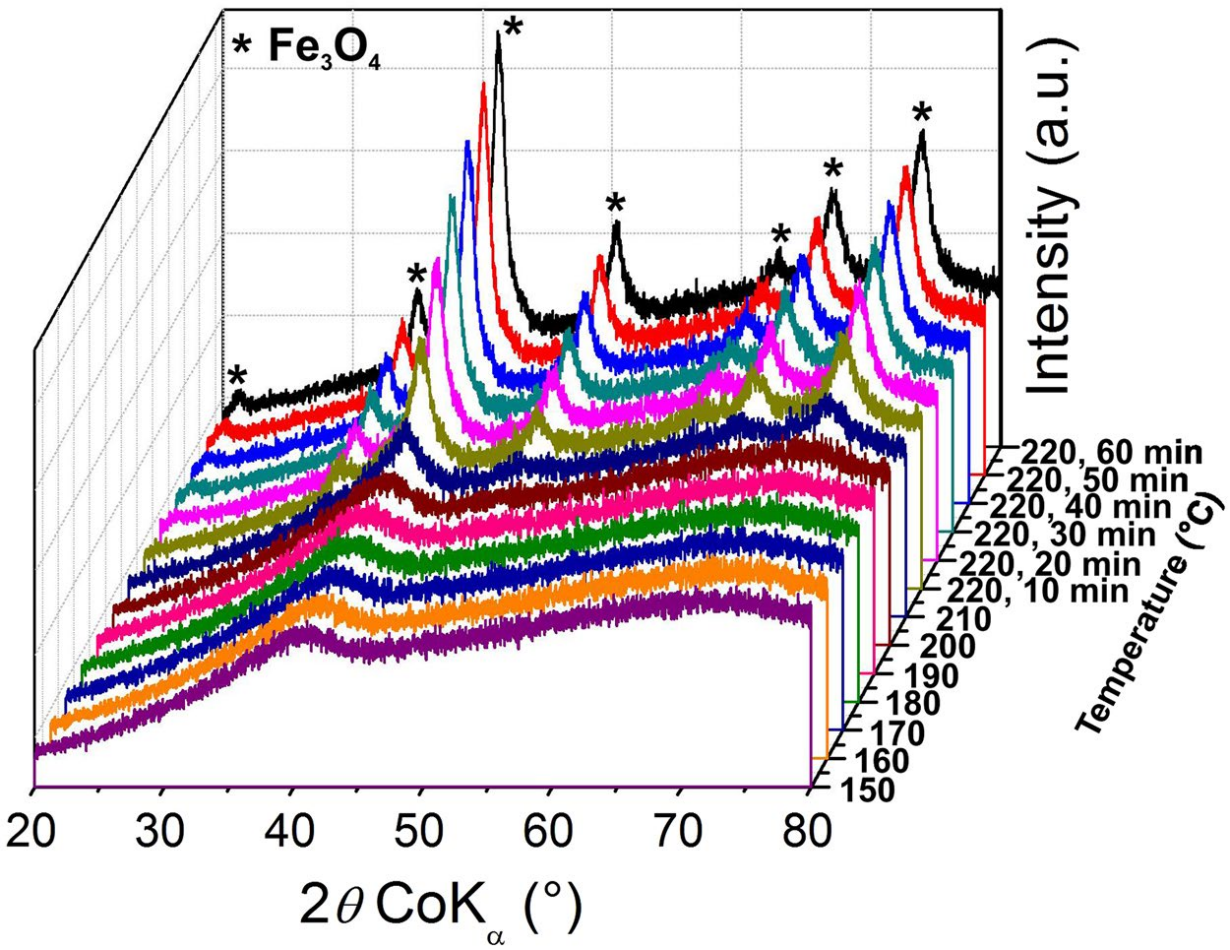
Evolution of X-ray diffraction patterns during *in situ* monitored thermally induced transformation of iron(III) oxide with ultra-small particles to magnetite in hydrogen gas atmosphere.

The preparation of magnetite microspheres by reduction of iron(III) oxide under a hydrogen atmosphere has been well investigated^40, 41^. Herein, we report a novel method for the synthesis of magnetite with a unique flower-like morphology from iron(III) oxalate *via* a simple two-step approach. Firstly, thermally induced solid state decomposition of iron oxalate was used to produce iron(III) oxide (Fe_2_O_3_) with ultra-small nanostructured particles; and secondly, the subsequent thermally induced reduction of the prepared iron(III) oxide under a hydrogen atmosphere afforded magnetite (Fe_3_O_4_). Figure 2 depicts the stepwise transformation of iron(III) oxide to magnetite by hydrogen reduction process *via in situ* monitoring by XRD. The two shoulders (around 40° and 74° of 2θ) are clearly visible in the diffraction patterns up to 210 °C confirming that the material is iron(III) oxide with ultra-small particles. At 220 °C, the diffraction lines belonging to *fcc* structure of magnetite/maghemite start to emerge and their intensities gradually increased during the 60 min period of isothermal treatment. Thus, we choose temperature 220 °C for 2 hours as the optimum condition for the preparation of magnetite from iron(III) oxide with ultra-small particles using a tube furnace under a hydrogen atmosphere.

**Figure 3:**
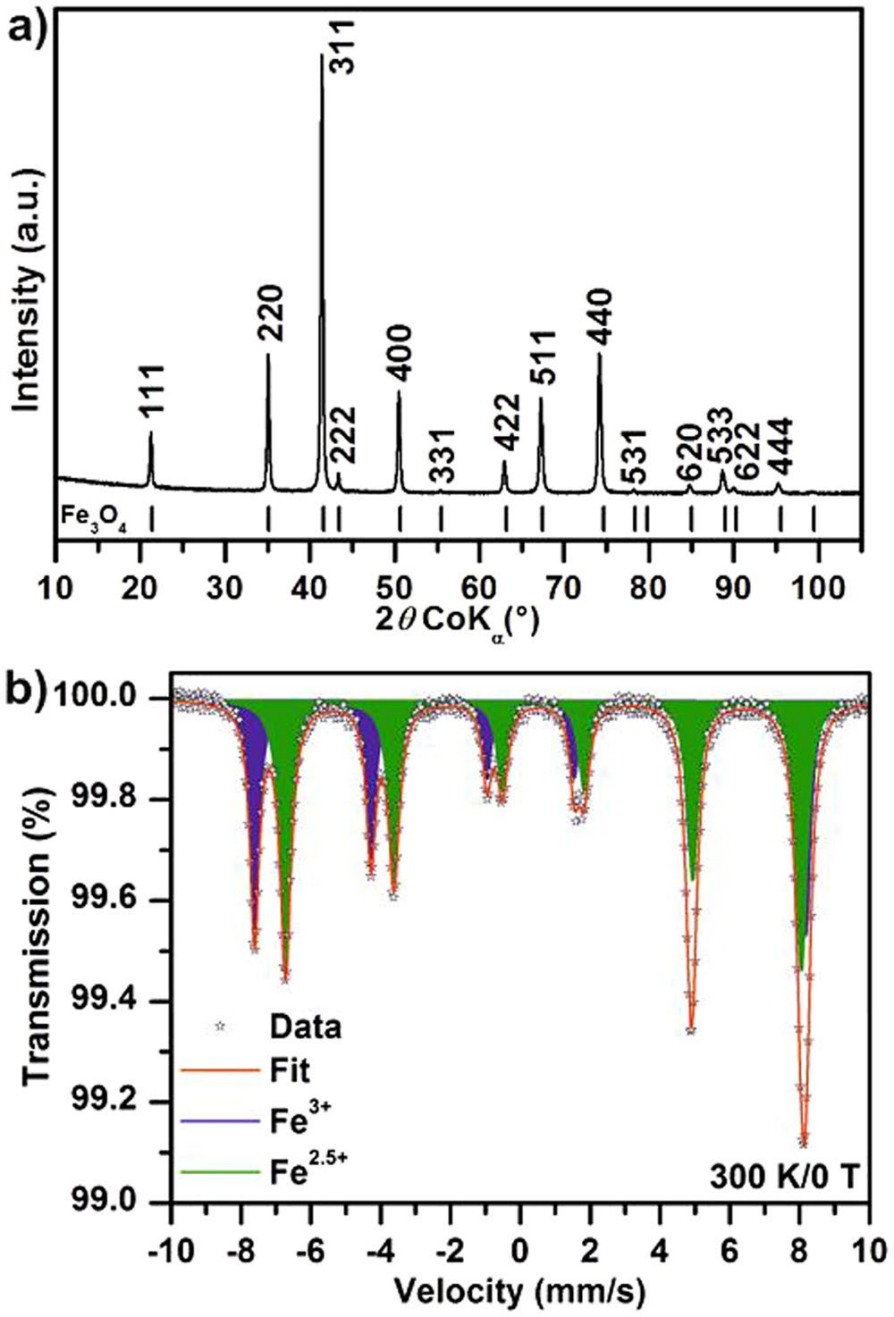
(**a**) XRD pattern and (**b**) Mössbauer spectrum of magnetite.

Figure 3a depicts XRD pattern of magnetite sample. All of the diffraction lines can be clearly ascribed to standard face-centered cubic (*fcc*) structure of Fe_3_O_4_ (space group: *Fd3m* (227), JCPDS card No. 01-089-3854). Although the isostructural character of magnetite and maghemite cause difficulties in direct and precise identification of these phases by XRD point of view, the cell parameter indicates correct suggestion; it varies from 0.8351 nm for maghemite and 0.8396 nm for stoichiometric magnetite^42, 43^. The cell parameter of cubic structure in the prepared sample is a = 0.8394 nm, which is in good agreement with values described for magnetite in the literature^44^. Nevertheless, the Mössbauer spectroscopy is a powerful experimental technique which provides precise identification of valence state of iron atoms and cations distribution and more specifically, for the identification of iron compounds. Consequently, Mössbauer spectroscopy was used for direct identification of iron oxide’s state (Fig. 2b). The acquired spectrum is composed of two magnetically split subspectra (i.e., sextets). The first sextet component with an isomer shift (*δ*) value of 0.27 mm s^−1^, quadrupole shift (*ε*_*Q*_) value of −0.01 mm/s and hyperfine magnetic field (B_*hf*_) value of 49.0 T corresponds to Fe^3+^ ions occupying all the tetrahedral positions in the Fe_3_O_4_ crystal structure and with a contribution from Fe^3+^ ions sitting in the octahedral sites having Fe^3+^ ions as the nearest neighbours (i.e. Fe^3+^–O–Fe^3+^ pathway). On the other hand, the second sextet with *δ* = 0.67 mm/s, *ε*_*Q*_ = 0.00 mm/s, and B_*hf*_ = 46.0 T is ascribed to Fe^2+^ and Fe^3+^ ions occupying the octahedral positions in the Fe_3_O_4_ crystal structure among which the electron hopping occurs (i.e., an Fe^2+^ ion with a neighbouring Fe^3+^ ion and *vice versa*; Fe^2+^–O–Fe^3+^ pathway) with a frequency faster than the characteristic time of the Mössbauer technique and thus manifested as a component with *δ* value lying in the range typical of an average valence state of + 2.5^45^. Relative spectral area of Fe^3+^ and Fe^2.5+^ sextet is 41 and 59%, respectively. This difference from ideal spectral area of 33 (Fe^3+^ sextet) and 67% (Fe^2.5+^ sextet) for stoichiometric magnetite indicates a nonstoichiometry in magnetite. In particular, Fe^3+^ ions having Fe^3+^ as the nearest neighbours in the octahedral sites, which thus do not participate in the electron hopping process, forms their own subspectrum with hyperfine parameters values very close to those of the subspectrum representing Fe^3+^ in the tetrahedral sites. Therefore, these two subspectra with nearly identical parameters are fitted as one and its relative area is increased to the detriment of subspectra representing mixed valence Fe^2.5+^ in octahedral sites.

**Figure 4:**
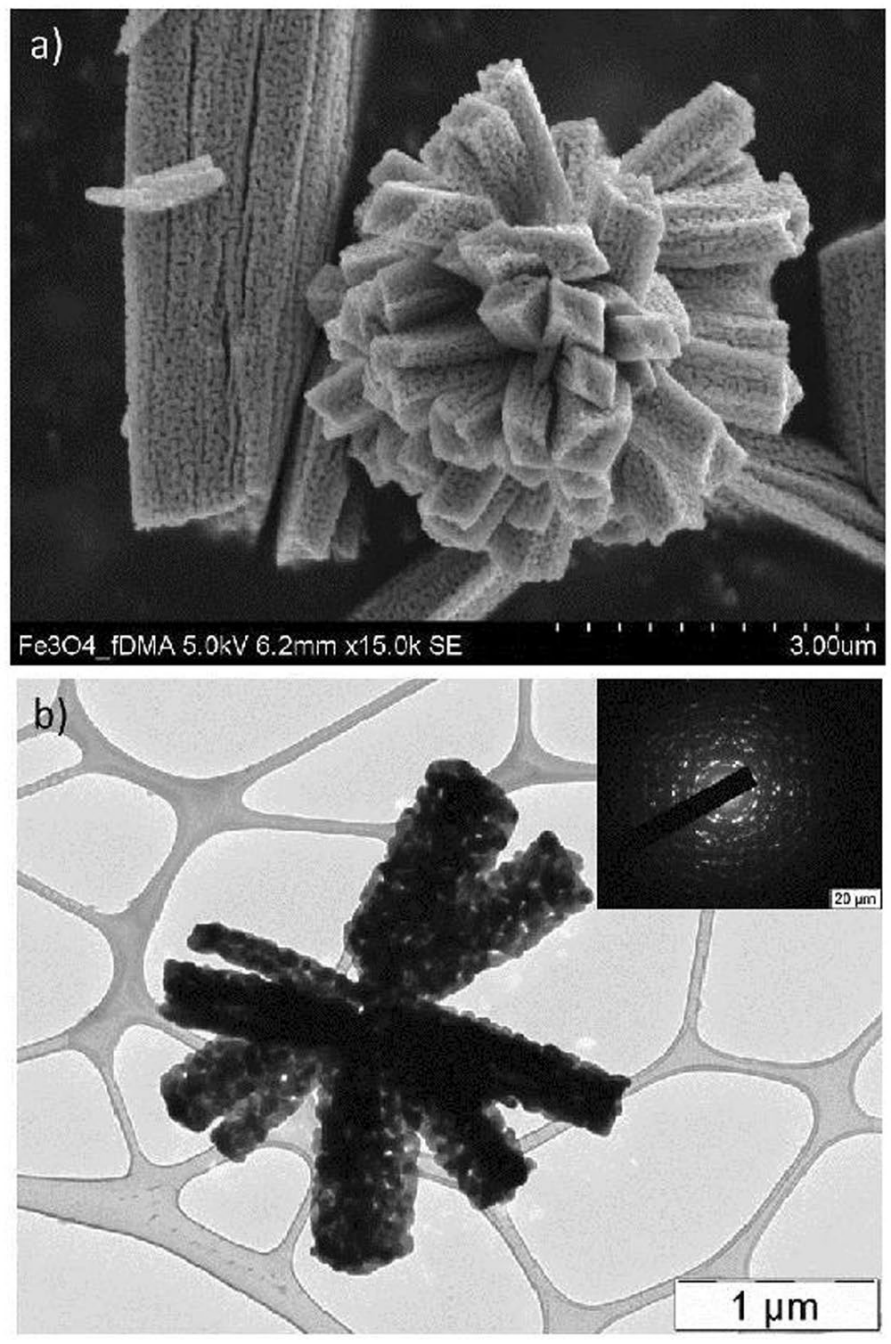
(**a**) SEM and (**b**) TEM image of magnetite.

The morphology of the prepared samples was obtained using scanning electron microscope (SEM) and transmission electron microscope (TEM). The SEM image (Fig. 4a) of magnetite revealed the retention of rod/flower like pattern as found in the case of iron(III) oxide^46^. From the TEM image (Fig. 4b), it can be seen that the individual nanorods possess an average breadth of size 300 nm while the self-assembled floral pattern has a diameter of about 3 μm.

TEM image (Fig. 4b) also showed the porous nature of the rods composed of interconnected microspheres. The sharp diffraction spot due to various planes of Fe_3_O_4_ in selected area electron diffraction (SAED) pattern (Fig. 4b inset) of the particles reveals well crystalline nature of materials. The width of the nanorods forming rod/flower like pattern was found to be uniform along its entire length as evidenced from the TEM image. The pores might be formed during recrystallization process or from the elimination of water during the reduction process.

**Figure 5:**
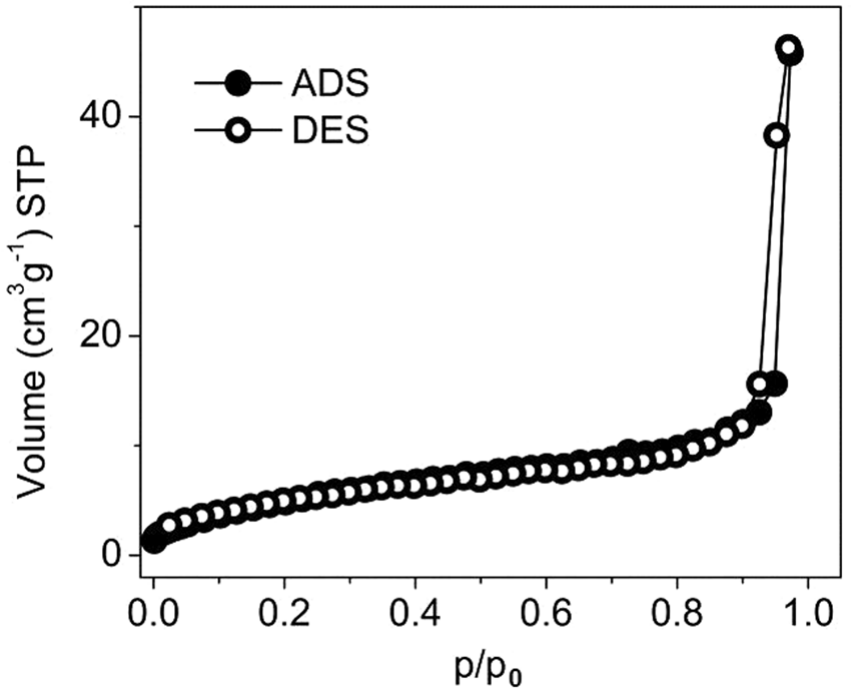
N_2_ adsorption-desorption isotherm of magnetite.

The N_2_ adsorption–desorption isotherms show Type II isotherm for the magnetite with a small hysteresis (Fig. 5) which reveal macroporous nature with cylindrical pores. The specific surface area obtained from BET method is 20 m^2^/g﻿.

Furthermore to examine the efficiency of the catalyst, we evaluated its reduction prowess for a variety of nitro compounds  to their corresponding industrially important amine derivatives in ethanol under microwave (MW) irradiation. Initially, to optimize the reaction conditions, various parameters such as effect of temperature, catalyst loading, solvent, and different hydrogen source including the amount of hydrazine hydrate were studied by choosing nitrobenzene as a model substrate. As expected, no reaction occurred in the absence of magnetite and hydrazine hydrate (Table 1, entries 1–3). Firstly, the reaction was carried out under conventional heating condition using magnetite (30 mg) as a catalyst and 150 μL hydrazine hydrate as hydrogen source in ethanol at 90 °C; complete conversion occurred in 3 h (Table 1, entry 14). Interestingly, when the reaction was performed under MW irradiation condition, the complete conversion could be achieved within 15 min (Table 1, entry 7) and no trace of substrate, intermediates or side product was evident by GC analysis. To explore the optimum amount of needed catalyst, different catalyst loadings (10, 20, and 30 mg) were investigated which revealed that 30 mg catalyst was the optimum loading that afforded > 99% conversion of nitrobenzene (Table 1, entry 5). The quantity of hydrazine hydrate did impact the conversion rate; reaction using 60 μL resulted in only 90% conversion (Table 1, entry 9), while 100 μL delivered quantitative conversion (Table 1, entry 7).

**Table 1:** Magnetite catalyzed catalytic reduction of nitrobenzene under microwave irradiation^a^


Entry	Catalyst	Amount of catalyst (mg)	Hydrazine hydrate (μL)	Temp. (˚C)	Time (Min)	^b^Conversion(%)	^b^Yield (%)
1	----	----	-----	90	30	0	0
2	-----	----	150	90	30	0	0
3	Fe_3_O_4_	30	----	90	30	0	0
4	Fe_3_O_4_	10	150	90	30	>97	95
5	Fe_3_O_4_	30	150	90	30	>99	98
6	Fe_3_O_4_	30	150	90	15	>99	98
7	Fe_3_O_4_	30	100	90	15	>99	98
8	Fe_3_O_4_	20	100	90	15	93	89
9	Fe_3_O_4_	30	60	90	15	>90	87
10	Fe_3_O_4_	30	100	50	15	35	25
11	Fe_3_O_4_	30	100	70	15	72	66
12	Fe_3_O_4_	30	100	90	10	>94	91
13	Fe_3_O_4_	30	-----^c^	90	15	0	---- ^c^
14	Fe_3_O_4_	30	100	90	180	99	97^d^
15	Fe_3_O_4_	30	100	rt	360	----	----

^a^ **Reaction conditions**: Nitrobenzene (0.5 mmol), Hydrazine hydrate (100 µL), Fe_3_O_4_ (30 mg), EtOH (1.5 mL), Temp 90°C. ^b^Determined by GC using dodecane as an internal standard, ^c^Isopropyl alcohol, ^d^Conventional heating.

Next, the effect of temperature on reduction reactions was determined and at 50 °C, 35% conversion and at 70 °C, 72% conversion was observed (Table 1, entries 10, and 11); increasing the temperature to 90 °C, however, afforded quantitative conversion within 15 min (Table 1, entry 7). Time variation, a crucial factor, was investigated next for complete conversion; 10 minutes delivered 94% conversion and 91% yield (Table 1, entry 12). Notably, with isopropyl alcohol as a hydrogen donor, no reaction occurred (Table 1, entry 13). The catalyst could catalyse the reaction under conventional heating conditions as well; however, an extended reaction time was required up to 240 min (Table 1, entry 14). Further, we observed that the reaction did not occur at room temperature and heating was essential for accomplishing this reaction (Table 1, entry 15).

The effect of different solvents on the reduction of nitrobenzene was also investigated and it was discerned that ethanol, 2-propanol, and acetonitrile afforded good conversion and yields (Table 2, entries 1, 3, and 5). For THF, the efficiency of the reduction significantly decreased and only 4% conversion was obtained while a mixture of EtOH:H_2_O (1:1) showed moderate conversion (Table 2, entry 2).

**Table 2:** Evaluation of different solvents for the reduction of nitrobenzene^a^


Entry	Solvents	^b^Conversion (%)	^b^Yield (%)
1	Ethanol	>99	98
2	EtOH:H_2_O	80	74
3	ACN	95	92
4	THF	4	-
5	2-propanol	95	91

^a^ **Reaction conditions:** Nitrobenzene (0.5 mmol), Hydrazine hydrate (100 μL), Fe_3_O_4_ (30 mg), solvent (1.5 mL), temperature (90 °C), time (15 min). ^b^Determined by GC using dodecane as an internal standard.

Assorted iron catalysts for the reduction of nitrobenzene with hydrazine hydrate were also examined under the optimized conditions. Notably, commercial Fe powder, FeSO_4_.7H_2_O, FeCl_3_.6H_2_O (Table 3, entries 1, 2 and 4) did not show any activity under these conditions, while FeCl_3_.4H_2_O, Fe(acac)_3_ and magnetite exhibited 18%, > 99%, and > 99% conversion, respectively (Table 3, entries 3, 5, and 6). Although, as-prepared magnetite and Fe(acac)_3_ have the same conversion and selectivity, but due to the homogeneous nature of Fe(acac)_3_, it cannot be recycled which limits its applications. In contrast, magnetite is a heterogeneous catalyst and has shown superiority due to its magnetic separation property and importantly, the ease of recyclability.

**Table 3:** Comparative evaluation of different iron species for the reduction of nitrobenzene^a^


Entry	Catalyst	^b^Conversion (%)	^b^Yield (%)
1	Fe powder	0	0
2	FeSO_4_.7H_2_O	0	0
3	Fe(acac)_3_	>99	98
4	FeCl_3_.6H_2_O	0	0
5	FeCl_3_.4H_2_O	18	14
6	Fe_3_O_4_	>99	98

^a^ **Reaction conditions:** Nitrobenzene (0.5 mmol), Hydrazine hydrate (100 μL), catalyst (30 mg), EtOH (1.5 mL), temperature (90 °C), time (15 min). ^b^Determined by GC using dodecane as an internal standard.

These optimized reaction conditions were then applied to an array of selected substituted nitroarenes bearing additional reducible groups to ascertain the chemoselectivity aspect and wider scope of the catalyst (Table 4). In most of the cases, quantitative ( > 99%) conversion of the substrates to the desired amine derivatives occurred within 15 min.

**Table 4:** Catalytic reduction of nitro compounds^a^.


**Entry**	**Nitro compound**	**Product**	^a^ **Conversion %**	^b^ **Yield %**
1			>99	96
2			>99	96
3			>99	95
4			>96	92^c^
5			>97	96
6			93	92
7			>42	37^c^
8			>99	98
9			>97	94^c^
10			99	96^d^
11			99	96
12			99	96
13			99	96^e^
14			99	95^f^
15			99	95^c^

^a^ **Reaction conditions**: Nitrobenzene (0.5 mmol), Hydrazine hydrate (100 μL), Fe_3_O_4_ (30 mg), EtOH (1.5 mL), temperature (90 °C), time (15 min).

^b^Determined by GC using dodecane as an internal standard.

^c^reaction time (25 min),

^d^reaction time (20 min),

^e^reaction time (22 min),

^f^isolated yield.

It was observed that for 5-nitro-1H-indole, sterically hindered 1-methyl-2-nitrobenzene and 6-nitro-2,3-dihydrobenzo[1,4]dioxine, the reactions were completed in 25 min (Table 4, entries 4, 9, and 15), while 3-fluoro nitrobenzene 4-methoxy nitrobenzene, and 4-methyl nitrobenzene exhibited 99% conversion in 22 min (Table 4, entries 10, 13 and 14). The sole exception was 4-nitrobenzamide which showed 42% conversion in 25 min (Table 4, entry 7) presumably due to the polar nature of the amidic compound. Interestingly, halogenated nitroarenes such as 2-chloro-4-iodo-1-nitrobenzene, 3-fluoro nitrobenzene, 4-bromo nitrobenzene, and 4-chloro nitrobenzene showed excellent conversions (Table 4, entries 1, 10, 11, and 12) without any dehalogenated product being observed. Easily reducible ester groups were well accommodated in this catalytic system (Table 4, entries 5 and 6). The catalytic prowess became apparent in the reduction of 4-nitrobenzonitrile, 6-nitroquinoline, and methyl (4-nitrophenyl)sulfane with excellent yield of the corresponding desired products (Table 4, entries 2, 3, and 8).

**Figure 6:**
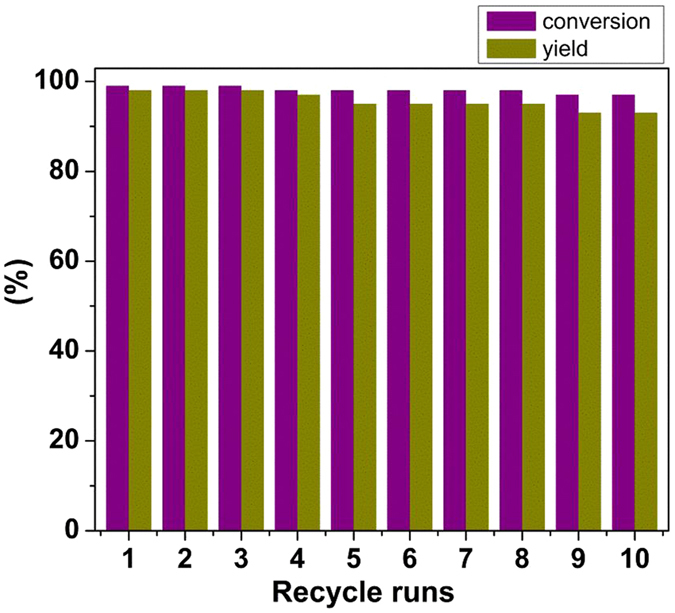
Reaction conditions: Nitrobenzene (1 mmol), Hydrazine hydrate (200 µL), Fe_3_O_4_ (60 mg), EtOH (3 mL), temperature (90 °C), MW. Determined by GC using dodecane as an internal standard.

The catalyst recycling is certainly very essential in heterogeneous catalytic reactions. Therefore, we examined the recyclability of our developed catalyst for reduction reaction by using nitrobenzene as a model substrate under the optimized conditions. After completion of the reaction, the catalyst could be easily separated using an external magnet. The separated spent catalyst was then washed with ethanol and dried before reuse. This process was repeated 10 times successfully without any noticeable decrease in catalytic activity (Fig. 6) suggesting that the catalyst could find application in the practical reduction of nitroarenes on industrial scale. The leaching aspect of any iron after recycling was examined by determining the metal content of reaction solution using AAS (atomic absorption spectroscopy) after removing of the catalyst; metal content was found to be 0.0735 ± 20% mg/L, which shows negligible leaching of iron from the catalyst which bodes well for its robustness and reusability.

**Figure 7:**
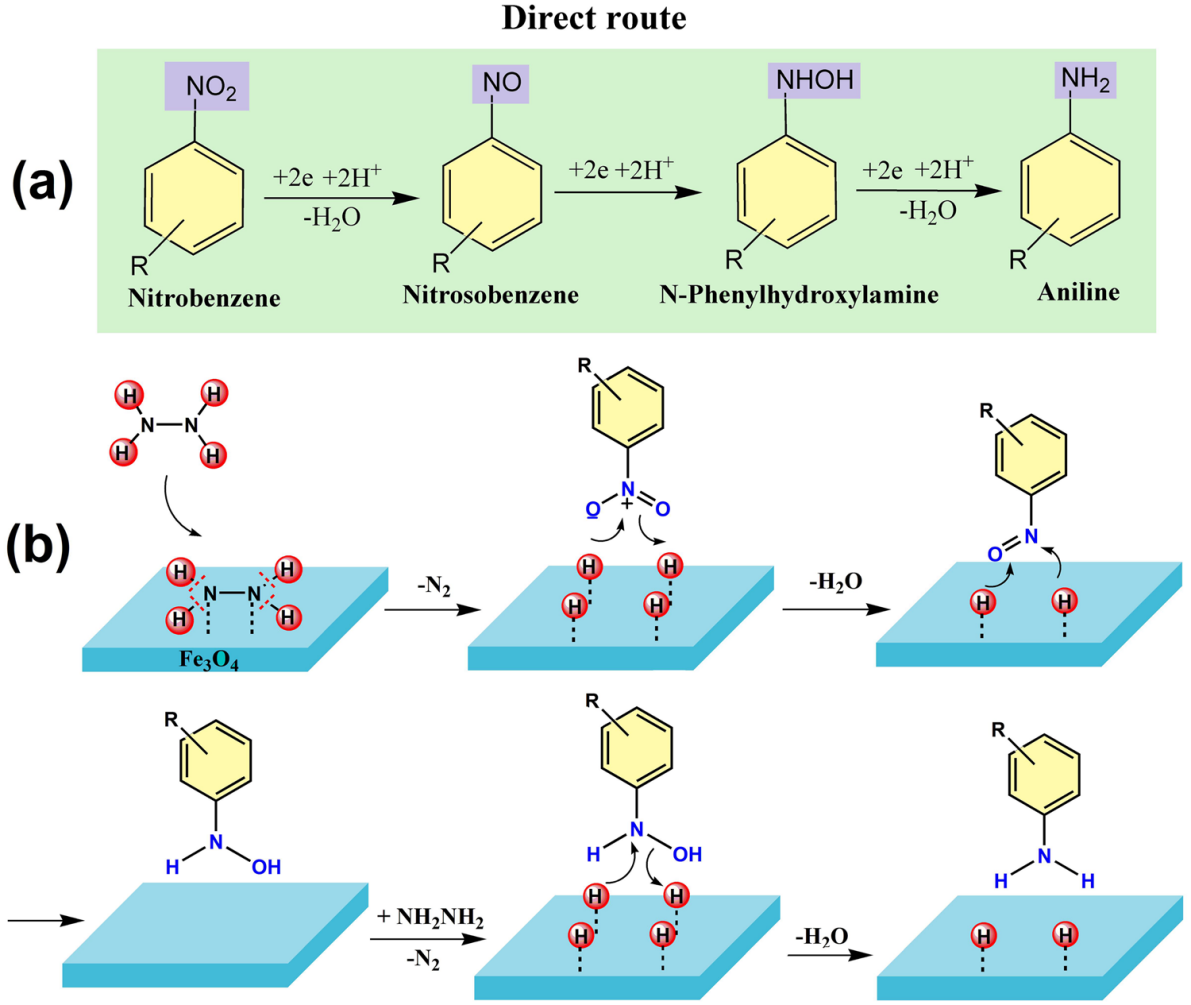
Schematics of (**a**) direct reaction route for reduction of nitroarene to anilines and (**b**) mechanism of nitroarenes reduction over the surface of magnetite *via* direct route using hydrazine hydrate as hydrogen source.

As mentioned in previous reports, that reduction of nitroarenes can proceed *via* two common routes^10, 47, 48^. The first direct route proceeds *via* nitrosobenzene and *N*-phenylhydroxylamine intermediates, (Fig. 7a). In contrast, the second route involves the condensation of nitrosobenzene and *N*-phenylhydroxylamine which advances through the intermediacy of azoxybenzene, azobenzene, and hydrazobenzene. In order to determine the exact route for this reduction of nitrobenzene, a reaction under identical reaction conditions was conducted for azobenzene. At the end of reaction, only hydroazobenzene could be isolated and no trace of aniline was detected which confirmed that reduction of nitroarene proceeded *via* first direct route.

In view of experimental validation of the direct route and on the basis of previous literature reports, a plausible mechanism is proposed^49–52^. The reaction initiates with the adsorption of hydrazine on the surface of magnetite nanoflowers followed by bond dissociation which produces nitrogen and surface-bound hydrogen as metal hydride. The nitroarenes adsorbed on the surface of magnetite thus get transformed to nitrosoarenes after reaction with surface adsorbed hydrogen. These highly active nitroso moieties further react with hydrogen to form stable hydroxylamine; hydrogenation of hydroxylamine is slow and the rate determining step. In the next step, two proton transfers produce the desired aniline derivatives (Fig. 7).

## Conclusion

In summary, we have established a robust, chemoselective and magnetically reusable catalyst for the reduction of industrially valuable nitroarenes substrates in the presence of other sensitive reducible functional groups. A diverse range of amines derivatives could be obtained expeditiously (15 min) in excellent yields under the MW heating conditions at 90 °C using hydrazine hydrate as a hydrogen source which precludes the use of a precious metal catalysts and hydrogen gas in the preparation of amines derivatives. The magnetite with a unique morphology prepared by our method was found to be very stable and could be used ten times successfully with minor decrease in its catalytic activity. The excellent catalytic performance, simple and a safe procedure, easy separation, and the recyclability make this environmentally benign catalytic system a remarkable and useful alternative to other Fe-based catalytic systems.

## Methods

### Materials

All solvents, hydrazine hydrate (50–60%), iron (II) chloride tetrahydrate (99.99%), oxalic acid (98%), *N*,*N*-dimethylacetamide (DMA) ( ≥ 99%), were purchased from Aldrich as analytical grade and were used without further purification.

### Preparation of the magnetite catalyst

In a typical synthesis protocol, 1 mmol (0.126 g) of oxalic acid (H_2_C_2_O_4_.2H_2_O) was dissolved in 10 mL of DMA under continuous magnetic stirring and admixed with an equal mole ratio (0.198 g) of aqueous iron chloride (FeCl_2_.4H_2_O) followed by addition of 12 mL deionised water. After stirring for 10 min, the as-obtained yellow coloured product (iron oxalate) was separated by centrifugation and washed with ethanol several times and dried at 333 K for 12 h. The as-prepared iron oxalate was thermally treated in air at the conversion temperature of 448 K for 12 h to obtain mesoporous iron(III) oxide (Fe_2_O_3_)^46^. Further, magnetite (Fe_3_O_4_) was prepared by thermally induced solid state reaction of iron(III) oxide in hydrogen gas at 220 °C for 2 h.

### General procedure for the reduction of nitrobenzene

Into a 10 mL microwave vial equipped with a magnetic stir bar, was placed 0.5 mmol of nitro compound in ethanol (1.5 mL), 100 μL of hydrazine hydrate followed by 30 mg catalyst. The vial was sealed with a Teflon-lined septum and irradiated with microwaves in a Monowave 300 single-mode MW reactor (Anton Paar GmbH, Graz, Austria) at 90 °C for 15 min. Progress of the reaction was monitored by TLC (silica gel; hexane/ethyl acetate) and the conversion and yield were determined by GC (gas chromatography) using *n*-hexadecane as an internal standard.

### Characterization

XRD patterns of materials were recorded on an X’Pert PRO diffractometer (PANanalytical) in Bragg-Brentano geometry with iron-filtered Co-Kα radiation (λ = 1.7903 Å) equipped with fast X’celerator detector. The reaction chamber XRK900 (Anton Paar) mounted to the diffractometer was employed for *in situ* monitoring of the preparation of the magnetite sample. Data were processed in High Score Plus Software in conjunction with PDF-4 + and ICSD databases.

The ^57^Fe Mössbauer Spectroscopy measurements were carried out to investigate iron-bearing phase compositions in the studied samples. Mössbauer spectra were recorded with 1024 channels and measured at room temperature employing MS2006 Mössbauer spectrometer based on virtual instrumentation technique^53, 54^, operating at a constant acceleration mode and equipped with a ^57^Co(Rh) source. The acquired Mössbauer spectra were processed (i.e., noise filtering and fitting) using the MossWinn software program^55^. The isomer shift values were referred against α-Fe foil sample at room temperature.

FESEM images were recorded on a Hitachi 6600 FEG microscope operating in the secondary electron mode and using an accelerating voltage of 5 kV. Detailed particle size and morphological studies of solid samples were performed by TEM on a JEOL JEM-2010 instrument equipped by a LaB_6_ cathode (accelerating voltage of 160 kV; point-to-point resolution of 0.194 nm). A drop of high-purity ethanol was placed onto a holey carbon film supported by a copper-mesh TEM grid (SPI Supplies, USA) and air-dried at room temperature. The dimensions of the microspheres were measured using ITEM software.

Nitrogen adsorption-desorption isotherms at 77.4 K were measured up to the saturation pressure of nitrogen (molecular cross-sectional area 0.162 nm^2^), and obtained by the static volumetric technique on an Autosorb-iQ-C analyzer (Quantachrome). Prior to the measurements, samples were degassed at room temperature for 12 h to reach pressure below 0.001 torr. Specific surface areas were calculated using the multipoint BET (Brunauer-Emmett-Teller) model. The best fits were obtained using adsorption data in the relative pressures of 0.08/0.25 (P/P_0_). The analysis and evaluations were performed with the ASiQwin 2.0 software package (Quantachrome). For the reaction, 10 mL glass vial equipped with Teflon-lined cap was irradiated in a Monowave 300 single-mode microwave reactor (Anton Paar GmbH, Graz, Austria) having auto adjusting MW power to maintain the reaction temperature.

The nitroarenes reduction products were analyzed using an Agilent 6820 GC equipped with an Agilent DB-5 capillary column (30 m × 0.32 mm, 0.5 m) under the operation parameters: inlet temperature of 100 °C, temperature of flame ionization detector of 250 °C, temperature ramp of the oven from 100 to 250 °C at a rate of 10 °C min^−1^.
